# Nurses’ knowledge, attitude and practice regarding non-pharmacologic interventions for behavioral and psychological symptoms of dementia

**DOI:** 10.1186/s12888-024-05962-2

**Published:** 2024-07-24

**Authors:** Hongdi Du, Xiaojing Wang, Xiaoxing Lai, Wei Wang, Xiaopeng Huo

**Affiliations:** 1 Department of health care, Peking Union Medical College Hospital, Chinese Academy of Medical Sciences, Beijing, China; 2 Nursing Department, Peking Union Medical College Hospital, Chinese Academy of Medical Sciences, Beijing, China

**Keywords:** Dementia, Behavioral and psychological symptoms of dementia, BPSD, Surveys and questionnaires, Health knowledge, attitudes, practice, Nurses

## Abstract

**Background:**

To evaluate the knowledge, attitude and practice of nurses regarding non-pharmacologic therapies for behavioral and psychological symptoms of dementia (BPSD).

**Methods:**

This cross-sectional, questionnaire-based study enrolled nurses at Peking Union Medical College Hospital (Beijing, China) between September 2022 and October 2022. Correlations between knowledge, attitude and practice scores were evaluated by Pearson correlation analysis. Factors associated with knowledge, attitude and practice scores were identified by multivariable linear regression. Based on a cross-sectional questionnaire survey, this study designed a questionnaire according to the Guidelines for Diagnosis and Treatment of Dementia in China, and randomly selected nurses from Peking Union Medical College Hospital to fill in the questions through the Wen-Juan-Xing online platform from September 2022 to October 2022.

**Results:**

The analysis included 210 nurses (202 females). The average knowledge, attitude and practice scores were 11.06±2.61 (total score: 18), 53.51±5.81 (total score: 60) and 64.66 ± 10.35 (total score: 80) points, respectively. Knowledge score was positively correlated with attitude score (*r* = 0.416, *P* < 0.001) and practice score (*r* = 0.389, *P* < 0.001); attitude and practice scores were also positively correlated (*r* = 0.627, *P* < 0.001). Multivariable analysis demonstrated that age ≥ 40 years-old (vs. ≤30 years-old) was associated with higher knowledge score (β = 1.48, 95% confidence interval [95%CI] = 0.42–2.54, *P* = 0.006). Age ≥ 40 years-old (β = 1.43, 95%CI = 0.35–2.51, *P* = 0.010 vs. ≤30 years-old) and bachelor’s degree or higher (β = 1.11, 95%CI = 0.12–2.10, *P* = 0.028 vs. college degree or lower) were associated with higher practice score.

**Conclusions:**

Older age and higher education level were associated with higher knowledge, attitude and/or practice scores. The findings of this study may help guide the development and implementation of education and training programs to improve the management of BPSD by nurses in China.

**Supplementary Information:**

The online version contains supplementary material available at 10.1186/s12888-024-05962-2.

## Background

Dementia is a global health issue that exerts substantial burdens on individuals, families and healthcare systems [1]. According to recent research [2], China currently holds the highest number of dementia patients globally. Furthermore, projections indicate that this population is expected to rise from approximately 16 million in 2020 to 49 million by 2050 [3].This potential increment has been associated to population aging and growth, and a rise in the prevalence of modifiable risk factors such as obesity, hyperglycemia and smoking [4].

Behavioral and psychological symptoms of dementia (BPSD) are experienced by most people with dementia at some stage during the course of their illness [5]. A cross-sectional study in China found that 50% of community-dwelling persons with dementia had BPSD [6]. BPSD encompasses a range of symptoms and behaviors such as anxiety, depression, delusions, aggression, agitation, wandering, sexual disinhibition and inappropriate social behavior [7]. BPSD is associated with an elevated risk of admission to a nursing home [8] and poorer prognosis [9]. Given that BPSD is a serious public health problem, it is important that it is diagnosed and treated in a timely manner.

The management of BPSD includes both pharmacologic and non-pharmacologic interventions [10]. Pharmacological interventions are often not the first-line approach for managing BPSD, primarily due to concerns related to side effects and increased mortality risk [11]. Elderly individuals, especially those with dementia, may be more sensitive to medication side effects, and cognitive decline can exacerbate these impacts [12]. Furthermore, while pharmacological interventions may offer short-term relief, their long-term efficacy in managing BPSD is often limited, making non-pharmacological approaches more favorable for sustained care [13]. A wide variety of non-pharmacologic interventions are available, including sensory stimulation, cognitive/emotion-oriented interventions, behavior management techniques, exercise therapy, animal-assisted therapy, special care units and dining room environment-based interventions [14, 15]. Nevertheless, the practical implementation of nonpharmacological strategies faces numerous challenges, primarily stemming from a shortage of trained personnel, limited understanding of the effectiveness of nonpharmacological interventions, staff opinions and preferences, and an expectation of fast resolution of symptoms [16]. In China, a recent review denoted that healthcare providers, both formal and informal, generally perceive their dementia knowledge and confidence in providing care as poor. Healthcare professionals and caregivers express concerns about the limited availability of specialized dementia education and training opportunities [17]. The appropriate utilization of non-pharmacologic therapies for BPSD requires that medical staff who take care of people with dementia are aware of the available options and avoid misconceptions that promote negative attitudes toward potentially useful treatments.

Knowledge, attitude and practice (KAP) surveys provide important information about the baseline knowledge, attitudes, beliefs, misconceptions and behaviors towards a health-related topic [18]. Furthermore, the information provided by these surveys can help in the design and implementation of public health programs to overcome issues and barriers to the use of an intervention [18]. Despite the significance and usefulness of understanding the KAP of healthcare providers, there is currently a scarcity of KAP studies about the treatment of dementia that is focused on the proficiency of nurses and considers only non-pharmacological approaches. Previous international [16, 19, 20] and Chinese [17, 21] reports have taken a comprehensive approach by considering both pharmacological and non-pharmacological therapies. However, these reports involved a combination of general practitioners, doctors, nurses, and other caregivers.

Due to their critical role as primary caregivers in managing BPSD, conducting a KAP survey targeting nurses specifically seems essential. The direct interaction of nurses with patients positions them to significantly influence patient outcomes in the implementation of non-pharmacologic interventions, which are recommended as first-line treatments [13]. Understanding the current state of nurses’ KAP can identify educational gaps and inform the development of targeted training programs. These insights are crucial for enhancing the competencies of nurses, improving patient care, and informing policy decisions within the specific cultural and healthcare context of China. Therefore, the aim of this study was to evaluate the KAP of Chinese nurses specifically with regard to non-pharmacologic therapies for BPSD.

## Methods

### Study design and participants

This descriptive correlational study enrolled nurses at Peking Union Medical College Hospital, Beijing, China between September 2022 and October 2022. The inclusion criteria were: (1) registered clinical nurse; (2) working in a geriatrics or dementia-related department; and (3) at least 1 year of clinical experience. This study was approved by the Medical Ethics Committee of Peking Union Medical College Hospital (JS-2917), all participants provided written informed consent. I confirm that all methods were performed in accordance with the relevant guidelines. All procedures were performed in accordance with the ethical standards laid down in the 1964 Declaration of Helsinki and its later amendments.

### Questionnaire design and data collection

The first draft of the questionnaire was designed in Chinese with reference to the Guidelines for Diagnosis and Treatment of Dementia in China [22] and previously published studies [7, 10, 23]. The first draft was modified according to the opinions of five senior doctors. While the primary aim of our study was to evaluate the KAP of nurses regarding non-pharmacologic interventions for BPSD, we also conducted initial psychometric property testing of our questionnaire. Forty-five questionnaires were administered to random selected nurses through convenience sampling to pre-test reliability, Cronbach’s α coefficient was 0.916, indicating excellent reliability (i.e., internal consistency), and the Kaiser-Meyer-Olkin (KMO) value was 0.896, suggesting that the internal consistency and reliability of the questionnaire were adequate. The reliability of the questionnaire was further validated with supplementary confirmatory factor analysis (Supplementary Fig. 1 and Supplementary Tables 1–2).

The final questionnaire included four aspects: demographic data, knowledge dimension, attitude dimension and practice dimension. The demographic data collected by the questionnaire included gender, age, education level, professional title, years of professional experience in a dementia-related department, type of hospital, type of department, proportion of patients with dementia admitted to the ward, BPSD nursing experience, and participation in BPSD training for dementia. The knowledge dimension included 18 questions, scoring 1 point for a correct answer and 0 for an incorrect or unclear response, totaling 0–18 points. The attitude dimension had 12 questions, using a five-point Likert scale (strongly agree: 5 points to strongly disagree: 1 point), except the 11th question, which was reverse-scored. The total score for attitude ranged from 12 to 60 points. The practice dimension had 16 questions, scored on a five-point Likert scale (always: 5 points to never: 1 point), with reversed scoring for questions 3 and 4, totaling 16–80 points.

Then, electronic questionnaires were created using Sojump online platform in China (https://www.wjx.cn/app/survey.aspx). And distributed to the study participants. A QR code was generated for the electronic questionnaire, allowing participants to log in and fill it out by scanning the QR code sent via WeChat. To ensure the quality and completeness of the questionnaire results, each IP address was restricted to submitting only one questionnaire, and all items were made mandatory and anonymous. The researcher provided assistance in answering queries from the study participants and checked all questionnaires for completeness, internal coherence, and reasonableness. Questionnaires with any contradictory logic or incomplete responses were deemed invalid.

### Sample size

According to Hair et al. [24], a minimum of one hundred respondents is generally recommended to ensure the robustness and reliability of multivariable logistic analysis. Thus, we employed a final sample size of 210 nurses based on the expected variability in knowledge, attitude, and practice scores and the need to achieve sufficient statistical power for detecting meaningful associations.

### Statistical analysis

Continuous variables are expressed as the mean ± standard deviation (SD) and compared between groups using Student’s t-test (two groups) or one-way analysis of variance (three or more groups). Categorical variables are expressed as frequency (percentage). Pearson correlation analysis was used to evaluate the correlations between the knowledge, attitude and practice scores. Multivariable linear regression analysis was used to identify factors associated with the knowledge, attitude and practice scores. Variables with *P* < 0.05 in the univariable analysis were entered into the multivariable analysis, and beta coefficients (β) and 95% confidence intervals (95%CIs) were calculated. All statistical tests were two-sided, and the difference was considered to be statistically significant when the *P*-value was less than 0.05. The analysis was performed using Stata 17.0 (StataCorp, College Station, TX, USA).

## Results

### Baseline characteristics

A total of 210 nurses (202 females, 96.19%) participated in the survey. The baseline characteristics of the respondents are shown in Table 1. In particular, professional titles were categorized as primary, intermediate, and advanced. Primary-level nurses were typically entry-level professionals with less experience, while intermediate-level nurses had more years of practice and often hold additional certifications. Advanced-level nurses possessed significant clinical experience and higher academic qualifications, often taking on leadership or specialized roles within the healthcare setting.

**Table 1:** Baseline characteristics

Variables	*n* (%)	Knowledge score		Attitude score		Practice score	
		Mean ± SD	*P*	Mean ± SD	*P*	Mean ± SD	*P*
**Aggregate score**	210 (100)	11.06 ± 2.61	-	53.51 ± 5.81	-	64.66 ± 10.35	-
**Age**			0.008		0.068		0.004
≤ 30 years-old	72 (34.29)	10.50 ± 2.79		52.24 ± 6.76		61.44 ± 10.89	
30–40 years-old	105 (50.00)	11.09 ± 2.40		54.26 ± 5.10		66.11 ± 9.39	
≥ 40 years-old	33 (15.71)	12.18 ± 2.54		53.91 ± 5.38		67.03 ± 10.69	
**Gender**			0.847		0.004		0.064
Male	8 (3.81)	10.88 ± 3.14		47.75 ± 6.90		58.00 ± 12.08	
Female	202 (96.19)	11.07 ± 2.60		53.74 ± 5.66		64.92 ± 10.22	
**Level of education**			0.018		0.003		0.013
College or below	35 (16.67)	10.11 ± 2.92		50.89 ± 5.96		60.71 ± 11.31	
Bachelor’s degree or above	175 (83.33)	11.25 ± 2.51		54.03 ± 5.65		65.45 ± 10.00	
**Professional title**			0.150		0.006		0.165
Without professional title	8 (3.81)	10.25 ± 2.12		47.50 ± 6.19		58.63 ± 6.41	
Primary	107 (50.95)	10.79 ± 2.70		53.35 ± 5.99		64.29 ± 10.69	
Intermediate/advanced	95 (45.24)	11.43 ± 2.51		54.20 ± 5.31		65.58 ± 10.10	
**Working years**			0.980		0.375		0.759
Less than 5 years	87 (41.43)	11.10 ± 2.69		53.08 ± 5.91		65.09 ± 9.03	
5–10 years	66 (31.43)	11.05 ± 2.63		53.29 ± 6.33		63.88 ± 11.56	
More than 10 years	57 (27.14)	11.02 ± 2.50		54.42 ± 4.97		64.89 ± 10.88	
**Type of hospital**			0.252		0.042		0.721
General hospital	175 (83.33)	11.15 ± 2.64		53.87 ± 5.69		64.77 ± 10.05	
Specialized hospital	35 (16.67)	10.60 ± 2.42		51.69 ± 6.14		64.09 ± 11.86	
**Type of ward**			0.771		0.711		0.836
General geriatric ward	190 (90.48)	11.08 ± 2.64		53. 56 ± 5.85		64.71 ± 10.38	
Dementia ward	20 (9.52)	10.90 ± 2.40		53.05 ± 5.51		64.20 ± 10.29	
**Proportion of patients with dementia admitted to ward**			0.918		0.678		0.758
< 50%	138 (65.71)	11.12 ± 2.64		53.43 ± 5.53		64.97 ± 10.01	
50–75%	45 (21.43)	10.96 ± 2.63		53.22 ± 6.23		63.64 ± 11.17	
> 75%	27 (12.86)	10.96 ± 2.50		54.41 ± 6.63		64.74 ± 10.92	
**Experience caring for patient with BPSD**			0.039		0.002		0.111
Yes	161 (76.67)	11.27 ± 2.48		54.20 ± 5.29		65.29 ± 10.25	
No	49 (23.33)	10.39 ± 2.93		51.24 ± 6.86		62.59 ± 10.50	
**Previous BPSD training**			0.210		0.024		0.029
Yes	89 (42.38)	11.33 ± 2.52		54.56 ± 5.24		66.47 ± 10.40	
No	121 (57.62)	10.87 ± 2.67		52.74 ± 6.10		63.32 ± 10.15	

BPSD: behavioral and psychological symptoms of dementia; SD: standard deviation

### Knowledge, attitude and practice scores

The average knowledge score was 11.06 ± 2.61 points (total: 18 points). The proportion of nurses giving correct answers to each of the 18 questions in the knowledge dimension ranged from 50.48 to 97.62% (Supplementary Table 2). Nurses aged ≥ 40 years-old (*P* = 0.008), those with a bachelor’s degree or higher (*P* = 0.018), and those with previous experience caring for patients with BPSD (*P* = 0.039) had significantly higher knowledge scores (Table 1).

The average attitude score was 53.51 ± 5.81 (total: 60 points). The distributions of the responses to the 12 questions in the attitude dimension are illustrated in Supplementary Fig. 2. As shown in Table 1, significantly higher attitude scores were observed for females (*P* = 0.004), those with a bachelor’s degree or higher (*P* = 0.003), nurses with a professional title (*P* = 0.006), those working in a general hospital (*P* = 0.042), nurses with experience caring for patients with BPSD (*P* = 0.002), and those who had received training in BPSD (*P* = 0.024).

The practice score for the respondents averaged 64.66 ± 10.35 (total: 80 points). The distributions of the responses to the 16 questions in the practice dimension are illustrated in Supplementary Fig. 3. Nurses aged ≥ 40 years-old (*P* = 0.004), those with a bachelor’s degree or higher (*P* = 0.013), and nurses who had received training in BPSD (*P* = 0.029) had significantly higher practice scores (Table 1).

### Correlations between the knowledge, attitude and practice scores

Pearson correlation analysis revealed that the knowledge score was significantly positively correlated with the attitude score (*r* = 0.416, *P* < 0.001) and knowledge score (*r* = 0.389, *P* < 0.001). There was also a positive correlation between the attitude and practice scores (*r* = 0.627, *P* < 0.001, Table 2).

**Table 2:** Correlation analysis of the three dimensions of the questionnaire

	Knowledge	Attitude	Practice
**Knowledge**	1		
**Attitude**	0.416*	1	
**Practice**	0.389*	0.627*	1

* *P* < 0.001

### **Factors associated with the knowledge**,** attitude and practice scores**

Multivariable analysis demonstrated that age ≥ 40 years-old (vs. ≤30 years-old) was significantly and independently associated with a higher knowledge score (β = 1.48, 95%CI = 0.42–2.54, *P* = 0.006). No variables were significantly related to attitude score, although a borderline significant result was obtained for age ≥ 40 years-old (*P* = 0.06). Age ≥ 40 years-old (β = 1.43, 95%CI = 0.35–2.51, *P* = 0.010 vs. ≤30 years-old) and bachelor’s degree or higher (β = 1.11, 95%CI = 0.12–2.10, *P* = 0.028 vs. college degree or lower) were significantly associated with a higher practice score (Table 3).

**Table 3:** Multivariable analysis of factors associated with knowledge, attitude and practice scores

Variable	Knowledge		Attitude		Practice	
	β (95%CI)	*P*	β (95%CI)	*P*	β (95%CI)	*P*
**Age**						
≤ 30 years-old	Ref.		Ref.		Ref.	
30–40 years-old	0.34 (-0.47, 1.14)	0.411	0.17 (-0.84, 1.19)	0.736	0.31 (-0.50, 1.12)	0.456
≥ 40 years-old	1.48 (0.42, 2.54)	0.006	1.25 (-0.03, 2.52)	0.056	1.43 (0.35, 2.51)	0.010
**Gender**						
Male			Ref.			
Female			-0.10 (-2.08, 1.88)	0.921		
**Education level**						
College or below	Ref.		Ref.		Ref.	
Bachelor’s degree or above	0.92 (-0.07, 1.91)	0.067	0.77 (-0.32, 1.87)	0.166	1.11 (0.12, 2.10)	0.028
**Professional title**						
Without professional title			Ref.			
Primary			-0.08 (-2.07, 1.91)	0.935		
Intermediate/advanced			0.22 (-1.99, 2.42)	0.844		
**Type of hospital**						
General hospital			Ref.			
Specialized hospital			-0.78 (-1.90, 0.33)	0.167		
**BPSD care experience**	Ref.					
No	0.66 (-0.17, 1.49)	0.118	Ref.			
Yes			0.67 (-0.21, 1.55)	0.135		
**BPSD training**						
No			Ref.		Ref.	
Yes			0.41 (-0.37, 1.20)	0.301	0.40 (-0.32, 1.12)	0.272

For each dimension score, only variables with *P* < 0.05 in the univariable analysis were entered into the multivariable analysis. BPSD: behavioral and psychological symptoms of dementia; 95%CI: 95% confidence interval

## Discussion

A notable finding of this study was that knowledge, attitude and/or practice scores were higher in nurses who were aged ≥ 40 years-old, had a bachelor’s degree or higher, had previous experience caring for patients with BPSD, or had received training in BPSD. Furthermore, the knowledge, attitude and practice scores were significantly correlated with each other. Additionally, age ≥ 40 years-old was independently associated with a higher knowledge score, attitude score (borderline-significant) and practice score, while having a bachelor’s degree or higher was independently associated with a higher practice score. To our knowledge, this is the first survey evaluating the knowledge, attitudes and practice of nurses regarding non-pharmacologic interventions for BPSD. Our findings provide new insights into the knowledge, attitudes and practices of nurses in China with regard to BPSD, and the results may help to inform the design and development of interventions to support nurses in the management of BPSD.

Although published research has assessed the knowledge, attitude and practice of general practitioners regarding BPSD and its management [16, 25, 26], there have been few KAP surveys of nurses [16, 20]. In the present study, the proportion of nurses correctly answering each of the 18 questions in the knowledge dimension ranged from 50.48% for statement 3 (“the assessment tools for BPSD in dementia”) and statement 18 (“pharmacologic intervention for BPSD is better than non-pharmacologic intervention, with less adverse reactions”) to 97.62% for statement 1 (“mental and behavioral symptoms are the changes in behavior, cognition, thought content and emotion of patients with dementia that occur during disease progression”). On the other hand, the question with the lowest correct rate was statement 8 (“Simulated existential therapy and nostalgia therapy are the same non-pharmacologic interventions”). Questions 7–16, which related to specific types of non-pharmacologic therapy, were answered correctly by 52.38–79.52% of the respondents, implying that there was room for improvement in the nurses’ knowledge of the various options available. The average knowledge score was approximately 11 points out of a maximum of 18 points, suggesting that the nurses in this study had low-to-moderate knowledge of non-pharmacologic interventions for BPSD. These findings are consistent with the deficit in knowledge about care of dementia patients among nurses globally, which was evidenced by an international systematic review [27]. Effective care and management have the potential to slow the progression of dementia in patients, enhancing their quality of life, prolonging lifespan, and easing the burden on caregivers [28]. Developed nations have implemented comprehensive dementia care models. The identified knowledge gaps in this study underscore the urgent need to promptly address the challenge of developing a dementia care and management model tailored to the specific conditions in China. Integrating dementia care into the standard curricula of nurse education may have the potential to improve the levels of knowledge. Although no previous surveys have specifically evaluated KAP of nurses regarding non-pharmacologic interventions for BPSD, general surveys relating to dementia or BPSD have been administered to caregivers by other researchers. For example, a lack of knowledge was suggested to contribute to the finding that a majority of surveyed general practitioners lacked confidence in the management of dementia and BPSD [16, 29, 30]. We suggest that the implementation of educational interventions, such as those described previously [31–34], may help to improve nurses’ knowledge of non-pharmacologic interventions for BPSD.

The mean attitude score of approximately 54 out of a maximum of 60 indicates that, overall, the participants in this study had a strongly positive attitude toward non-pharmacologic interventions for BPSD. The vast majority of respondents strongly agreed or agreed with the correct statements in 11 of the 12 questions in the attitude dimension (see Supplementary Fig. 1). The correct statements with the highest rates of strong agreement (77%) were number 6 (“I think besides clinical intervention, we should also pay attention to the support of patient’s families”) and 9 (“I think it is necessary to carry out health education on BPDS-related knowledge for caregivers”). However, only 49% of the nurses disagreed with the incorrect statement that it was unnecessary to use non-pharmacologic interventions to treat BPSD (question 11); this is not consistent with the consensus statement of the Academy of Cognitive Disorders of China [28], which states that BPSD should first be treated with non-pharmacologic therapies. In foreign studies, resistance to discontinuing medication therapy has been also reported among general practitioners, who attribute this attitude to a shortage of nursing staff and limited resources [35]. On the other hand, it has been found that nurses prefer managing behaviors non-pharmacologically [36]. In agreement with our findings, a recent survey of nurses in acute care settings also found that the nurses tended to have a very positive attitude but lacked some of the knowledge and skills needed to identify, prevent and manage BPSD [37]. Moreover, a systematic review concluded that positive attitude toward patients with dementia was found to be context and training related [27]. These factors of context and training could be the reason behind discrepancies in the attitude results among different studies. The practice score averaged around 65 out of a maximum of 80, indicating that there was some room for improvement in the practices of the nurses included in this research. Most of the respondents strongly agreed or agreed with the correct statements in 14 of 16 questions in the practice dimension. The statement with the highest level of agreement and strong agreement was number 2 (“When BPSD causes serious consequences, I will promptly inform the doctor to take drug intervention”). However, less than 40% of the respondents strongly disagreed or disagreed with incorrect statements regarding when to stop non-pharmacologic interventions for BPSD, revealing that some of the nurses may be inappropriately withdrawing these treatments in patients with BPSD. Again, these findings disagree with foreign reports of a preference of nurses toward non-pharmacological treatments [36]. On the other hand, our findings that higher education are associated with better practice scores are in agreement with previous findings [38].

Older age and higher education level were associated with higher knowledge and practice scores in our analysis. Prior experience and training in BPSD showed a trend towards higher scores. These findings suggest that experience and training may impact the knowledge, attitudes, and practices of nurses caring for patients with BPSD. In agreement with our results, a survey of Australian healthcare workers found that university education to at least bachelor level, previous dementia education experience, and time spent providing professional dementia care to patients were factors associated with knowledge of dementia [39]. Furthermore, dementia competence was better in nurses who were older, had more seniority, had more experience of caring for people with dementia, or had received training in dementia care [37].

As this is the first study of its kind in China, we anticipate that these findings will help in the locally tailored design and implementation of strategies to improve the knowledge, attitudes and practices of nurses caring for patients with BPSD. Educational experiences internationally have been shown to improve nursing students’ knowledge of Alzheimer’s disease and attitudes toward people with Alzheimer’s disease, including in the specific area of BPSD [40]. In addition to the development of educational programs, optimization of staffing patterns may help give nursing staff sufficient time to allow them to select and implement non-pharmacologic interventions for BPSD rather than drug-based treatments [41]. Policymaking in China could significantly enhance education regarding non-pharmacologic interventions for people with dementia by establishing a national dementia education and training strategy. This should include integrating dementia care into medical and nursing curricula and providing continuous professional development for healthcare workers. Such a policy would address the identified gaps in knowledge and training, thereby improving the competence and confidence of healthcare professionals in managing dementia. Additionally, developing culturally appropriate educational materials and training programs focused on non-pharmacologic interventions could help reduce stigma and improve attitudes towards dementia care. Furthermore, increasing support for family carers through community-based programs led by trained nurses could alleviate the burden on informal caregivers and ensure better care for dementia patients.

This study has some limitations. First, the sample size was not large, hence it cannot be excluded that the analysis lacked the statistical power to detect some real differences between groups. Second, this was a single-center study, so the generalizability of the findings remains unknown. Cultural, institutional, and regional differences in healthcare practices and patient demographics may influence the applicability of our findings to other settings. Third, although the KAP questionnaire was developed based on established tools, it may have limitations with regard to its ability to assess perceptions of BPSD. There is a possibility for self-reporting bias in the questionnaires, since external factors such as infrastructure and past experiences can significantly influence behavior, adding complexity to the interpretation of results. Fourth, what kind of patients (BPSD profiles, severity of dementia, usage of psychotropic drugs, etc.) did the nurse experienced, nurses’ native language, place of birth, personality traits, and intelligence quotient were not included as baseline characteristics, which may influence non-pharmacological approaches. A further limitation of our study is the predominance of female participants (96.19%), which may affect the generalizability of our findings. Future studies should aim to include a more balanced gender distribution to better understand the KAP of both male and female nurses in managing BPSD. Therefore, these limitations should be considered when conducting further analysis and interpretation.

While research focusing exclusively on nurses can provide valuable insights into their role in non-pharmacologic therapies for BPSD, there may be some potential drawbacks, particularly in terms of limiting a broader interdisciplinary understanding and collaboration. Limited perspective, missed opportunities for innovation, and limited generalizability of the results are also potential disadvantages of analyzing the KAP of nurses only. Non-pharmacologic therapies for BPSD should be approached collaboratively not only by nurses but also by other healthcare professionals such as pharmacists, psychologists, and physicians.

## Conclusions

In conclusion, older age, higher level of education, prior experience caring for patients with BPSD, and previous training in BPSD were factors associated with higher knowledge, attitude and/or practice scores. The results of our study yield importance insights into the knowledge, attitudes and practices of nurses in China regarding BPSD. We anticipate that the findings may help guide the development and implementation of education and training programs to improve the management of BPSD by nurses. However, more local studies are needed to elucidate and confirm the current KAP of nurses towards BPSD treatment in China.

### Electronic supplementary material

Below is the link to the electronic supplementary material.

Supplementary Material 1

## Data Availability

All data generated or analysed during this study are included in this published article.

## Abbreviations

BPSD: Behavioral and psychological symptoms of dementia
KAP: Knowledge, attitude and practice
KMO: Kaiser-Meyer-Olkin
