# Supplementary material for: Nurses’ knowledge, attitude and practice regarding non-pharmacologic interventions for behavioral and psychological symptoms of dementia
Source: BMC Psychiatry. 2024 Jul 24;24:528. doi: 10.1186/s12888-024-05962-2 (PMC11267692; doi:10.1186/s12888-024-05962-2)
Supplement: Supplementary file 1 — Supplementary Material 1 [file 12888_2024_5962_MOESM1_ESM.docx]

**Supplementary Table 1 Model Fitting of the Factor Analysis**

| Indicators | Reference | Results |
| --- | --- | --- |
| RMSEA | <0.08 Good | 0.060 |
| SRMR | <0.08 Good | 0.079 |
| TLI | >0.8 Good | 0.873 |
| CFI | >0.8 Good | 0.883 |

**Supplementary Table 2 Results of the Factor Analysis**

|  |  | Estimate | P>\|z\| |
| --- | --- | --- | --- |
| CK1 | Knowledge | 1 |  |
| CK2 | Knowledge | 2.09 | <0.001 |
| CK3 | Knowledge | 2.04 | 0.008 |
| CK4 | Knowledge | 1.44 | 0.001 |
| CK5 | Knowledge | 1.10 | 0.006 |
| CK6 | Knowledge | 1.63 | 0.001 |
| CK7 | Knowledge | 2.61 | 0.001 |
| CK8 | Knowledge | 0.84 | 0.187 |
| CK9 | Knowledge | 2.66 | 0.002 |
| CK10 | Knowledge | 0.32 | 0.617 |
| CK11 | Knowledge | 3.63 | <0.001 |
| CK12 | Knowledge | 3.48 | <0.001 |
| CK13 | Knowledge | 3.87 | <0.001 |
| CK14 | Knowledge | 1.00 | 0.135 |
| CK15 | Knowledge | -1.30 | 0.053 |
| CK16 | Knowledge | 1.91 | 0.003 |
| CK17 | Knowledge | 1.73 | 0.008 |
| CK18 | Knowledge | 1.41 | 0.055 |
| CA1 | Attitude | 1.00 |  |
| CA2 | Attitude | 0.96 | <0.001 |
| CA3 | Attitude | 1.11 | <0.001 |
| CA4 | Attitude | 1.00 | <0.001 |
| CA5 | Attitude | 1.11 | <0.001 |
| CA6 | Attitude | 1.05 | <0.001 |
| CA7 | Attitude | 1.08 | <0.001 |
| CA8 | Attitude | 1.19 | <0.001 |
| CA9 | Attitude | 1.03 | <0.001 |
| CA10 | Attitude | 1.03 | <0.001 |
| CA11 | Attitude | 0.33 | 0.152 |
| CA12 | Attitude | 0.96 | <0.001 |
| CP1 | Practice | 1.00 |  |
| CP2 | Practice | 1.00 | <0.001 |
| CP3 | Practice | -0.23 | 0.366 |
| CP4 | Practice | 0.07 | 0.78 |
| CP5 | Practice | 1.54 | <0.001 |
| CP6 | Practice | 2.01 | <0.001 |
| CP7 | Practice | 1.89 | <0.001 |
| CP8 | Practice | 1.95 | <0.001 |
| CP9 | Practice | 2.16 | <0.001 |
| CP10 | Practice | 2.13 | <0.001 |
| CP11 | Practice | 1.86 | <0.001 |
| CP12 | Practice | 2.19 | <0.001 |
| CP13 | Practice | 1.96 | <0.001 |
| CP14 | Practice | 2.09 | <0.001 |
| CP15 | Practice | 2.04 | <0.001 |
| CP16 | Practice | 1.81 | <0.001 |

**Supplementary Table 3. Responses to the items in the knowledge dimension.**

| **Knowledge** | **False rate** | **Correct rate** |
| --- | --- | --- |
|  | ***n* (%)** | ***n* (%)** |
| K1. Mental and behavioral symptoms are the changes in behavior, cognition, thought content and emotion of patients with dementia that occur during disease progression. | 5 (2.38) | 205 (97.62) |
| K2. According to the symptom group, BPSD can be divided into two types: mental symptoms and behavioral symptoms. | 28 (13.33) | 182 (86.67) |
| K3. The assessment tools for BPSD in dementia. | 104 (49.52) | 106 (50.48) |
| K4. The inducing factors of BPSD. | 17 (8.10) | 193 (91.90) |
| K5. The interventions for inducing factors of BPSD. | 15 (7.14) | 195 (92.86) |
| K6. The principles of non-pharmacologic intervention for BPDS. | 120 (57.14) | 90 (42.86) |
| K7. The music therapy for BPDS. | 146 (69.52) | 64 (30.48) |
| K8. Simulated existential therapy and nostalgia therapy are the same non-pharmacologic interventions | 148 (70.48) | 62 (29.52) |
| K9. Exercise therapy is effective for depressed mood, agitation, sleep disturbance, etc., but lacks evidence of efficacy for anxiety, apathy, and repetitive behaviors | 100 (47.62) | 110 (52.38) |
| K10. Pet therapy can reduce restlessness and improve the degree and quality of patients' social interactions but it cannot reduce the loneliness and anxiety of patients. | 140 (66.67) | 70 (33.33) |
| K11. Light therapy is suitable for patients with daytime rhythm disorder and "sunset syndrome" | 48 (22.86) | 162 (77.14) |
| K12. Cognitive behavioral therapy is mainly used to treat depression, anxiety and other psychological problems in BPSD | 59 (28.10) | 151 (71.90) |
| K13. Massage, craniosacral therapy and therapeutic touch are commonly used in touch therapy | 50 (23.81) | 160 (76.19) |
| K14. The common essential oils in aromatherapy. | 134 (63.81) | 76 (36.19) |
| K15. The music therapy belongs to sensory oriented therapy. | 143 (68.10) | 67 (31.90) |
| K16. The specific methods of cognitive oriented therapy. | 43 (20.48) | 167 (79.52) |
| K17. All methods of non-pharmacologic intervention are applicable to the patient without considering the individual situation of the patient | 53 (25.24) | 157 (74.76) |
| K18. Pharmacologic intervention for BPSD is better than non-pharmacologic intervention, with less adverse reactions | 104 (49.52) | 106 (50.48) |


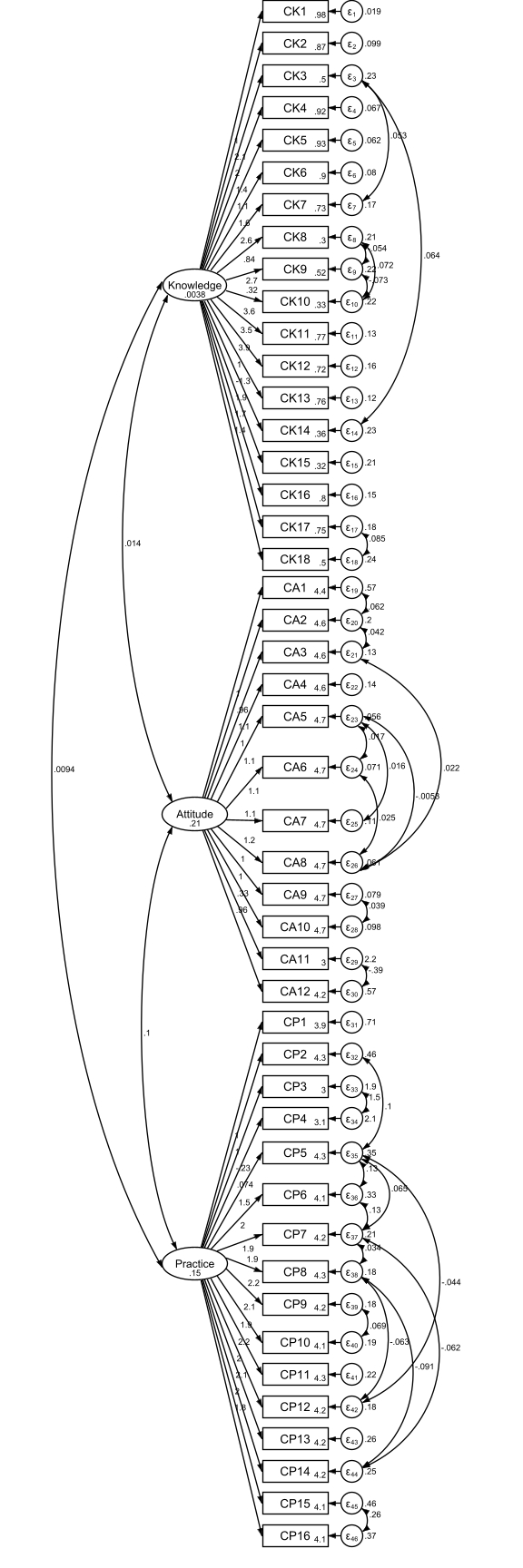


**Supplementary Figure 1. Illustrative Representation of the Factor Analysis**

**
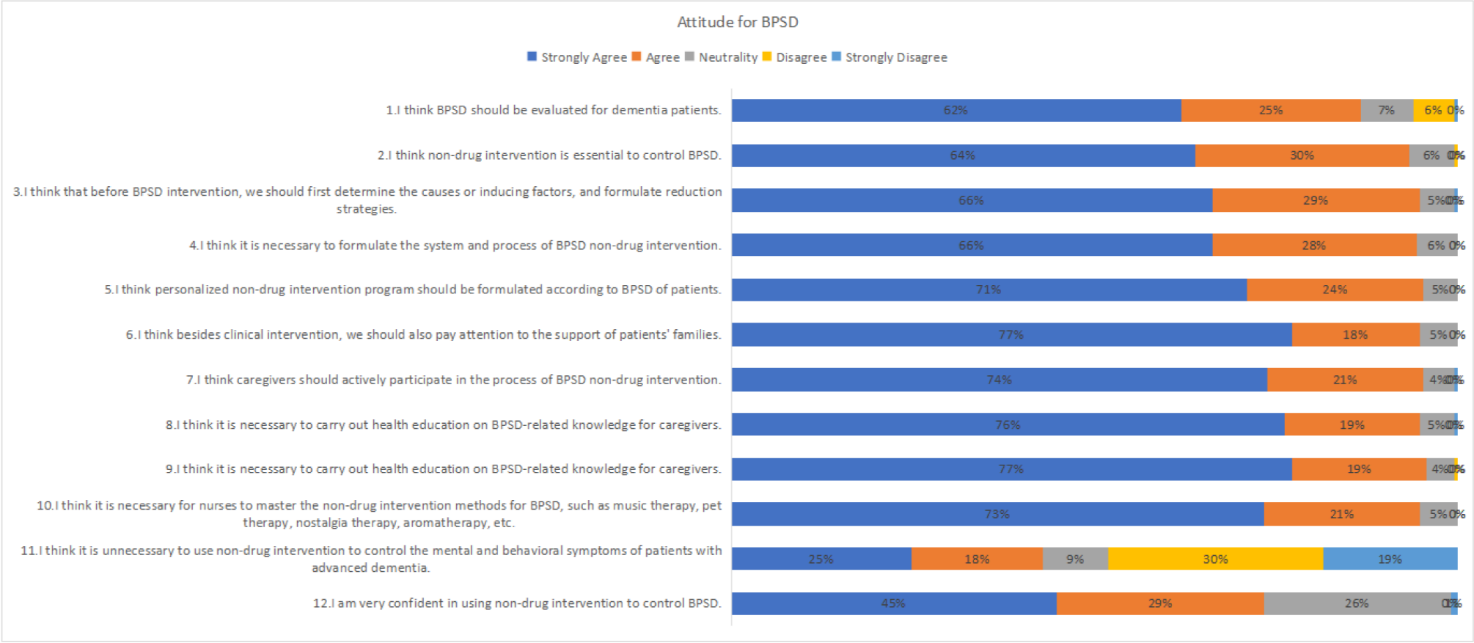
**

**Supplementary Figure 2. Distribution of responses for the attitude dimension.**

**
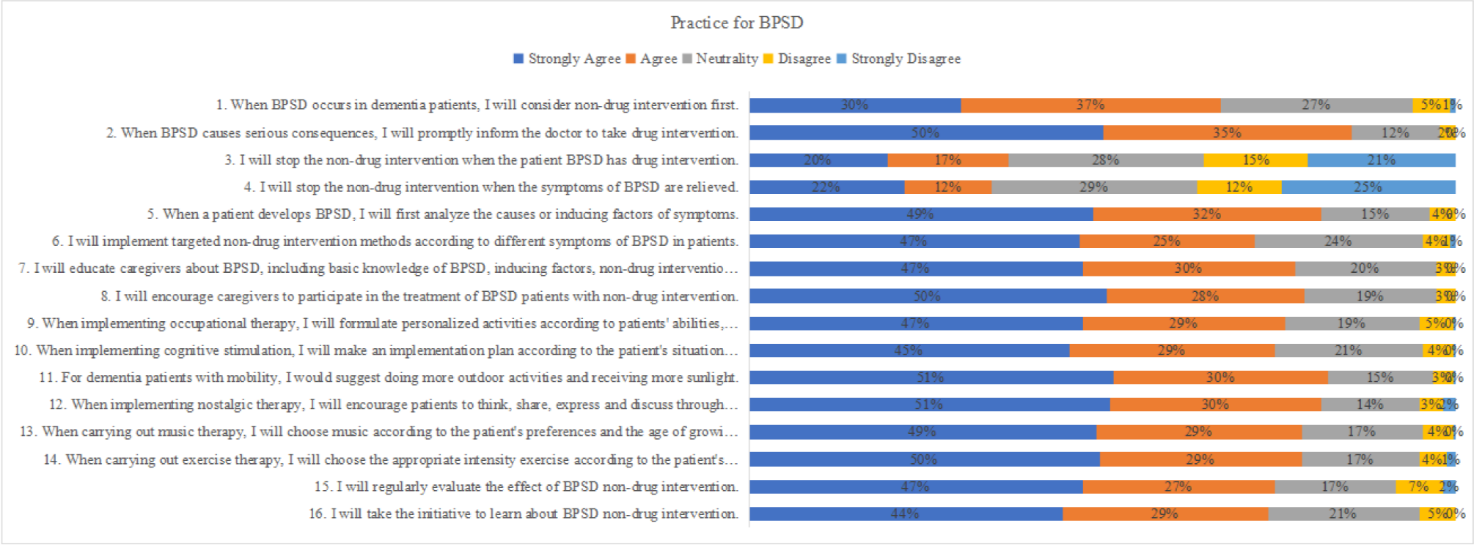
**

**Supplementary Figure 3. Distribution of responses for the practice.**
